# Emerging Therapies for Noninfectious Uveitis: What May Be Coming to the Clinics

**DOI:** 10.1155/2014/310329

**Published:** 2014-04-24

**Authors:** Jose R. Maya, Mohammad A. Sadiq, Liz J. Zapata, Mostafa Hanout, Salman Sarwar, Nithya Rajagopalan, Kathleen E. Guinn, Yasir J. Sepah, Quan Dong Nguyen

**Affiliations:** Ocular Imaging Research and Reading Center, Stanley M. Truhlsen Eye Institute, University of Nebraska Medical Center, 3902 Leavenworth Street, 985540 Nebraska Medical Center, Omaha, NE 68198-5540, USA

## Abstract

Corticosteroids along with other immunomodulatory therapies remain as the mainstay of treatment tor all patients with noninfectious uveitis (NIU). However, the systemic side effects associated with the long-term use of these drugs has encouraged the development of new therapeutic agents in recent times. This review article discusses upcoming therapeutic agents and drug delivery systems that are currently being used to treat patients with NIU. These agents mediate their actions by blocking specific pathways involved in the inflammatory process. Agents discussed in this review include full or recombinant monoclonal antibodies against interleukins such as IL-17 (secukinumab), IL-l (gevokizumab), and IL-6 (tocilizumab and sarilumab), antibody fragments against inflammatory cytokines such as TNF-**α** (ESBA 105) and T-cell inhibitors such as fusion proteins (abatacept), and next generation calcineurin inhibitors (voclosporin). In addition, administration of immune modulatory therapies using methods such as iontophoresis (EGP-437) and intravitreal injection (sirolimus) for the treatment of NIU' uveitis has also been discussed.

## 1. Introduction

Local and systemic corticosteroids are the mainstay of treatment for all patients with noninfectious uveitis (NIU); however, long term use of steroids can lead to both systemic and local adverse effects, such as cataracts, glaucoma, and metabolic disorders, among several others [1]. Increasing efforts are being made to develop a treatment option that will limit corticosteroid use and, therefore, decrease the risk of its associated adverse effects. Current guidelines recommend the addition of immunomodulatory therapy (antimetabolites, calcineurin inhibitors, alkylating agents, and tumor necrosis factor- (TNF-) alpha inhibitors) when inflammation cannot be controlled with ≤10 mg/day of prednisone within three months. Although this approach decreases the risks associated with corticosteroid use, immunomodulatory therapy (IMT) in itself has been associated with toxicities and has limited efficacy in some patients, further highlighting the need for a safer alternative to corticosteroids [2].

The index review article focuses primarily on the new therapeutic options for NIU, including novel agents and established drugs with innovative delivery systems.

## 2. Therapies in Development

### 2.1. AIN457 (Secukinumab)

IL-17 was first identified in rodent T-cell hybridoma and subsequently cloned in CD4 + T-cells in 1995. IL-17 is produced by TH17 cells and mediates its actions through a heterotrimeric receptor composed of two IL-17RA subunits and one IL-17RC subunit, consequently promoting the expression of antimicrobial peptides and inducing secretion of proinflammatory cytokines, chemokines, and metalloproteinases. New evidence suggests IL-17 activity in immune protection against parasites and viruses; however, in contrast to its protective role, it can also lead to adverse effects that result in tissue damage associated with various human inflammatory diseases such as rheumatoid arthritis (RA), psoriasis, multiple sclerosis (MS), and inflammatory bowel disease (IBD) [3]. Likewise in uveitis, the upregulation of IL-17A in patients with active Adamantiades-Behçet and Vogt-Koyanagi-Harada (VKH) diseases has led to the targeting of this interleukin in ocular inflammatory diseases [4, 5].

By blocking the pathogenic driver IL-17A, the fully human antibody AIN457 (Novartis Pharmaceutical, Basel, Switzerland) has been shown to interrupt inflammation in patients with RA, psoriasis, and NIU [6]. In an open label study of the safety and tolerability of secukinumab, 16 patients with active chronic NIU were treated with two infusions of AIN457 (10 mg/kg), at baseline and 3 weeks later. The majority of patients responded with a rapid reduction in vitreous haze that was sustained in the following 8 weeks with an increase of visual acuity (VA). No serious adverse events were reported [6]. Following the results of this study, further clinical trials have been initiated to evaluate the efficacy and safety of secukinumab in NIU. Dick et al. recently reported a significant reduction in mean total postbaseline immunosuppressive medication (ISM) scores with no loss in visual acuity (VA) in patients treated with AIN457 for NIU. However, the primary endpoint of the study, that is, the uveitis recurrence in patients receiving secukinumabcompared to the placebo group, was not statistically significant in any study. Secukinumab was associated with a significant reduction in mean total postbaseline ISM score (*P* = 0.019; 300 mg q4w versus placebo) in the SHIELD study.

Likewise, secukinumab was associated with a greater median reduction in ISM score versus placebo in the INSURE study, although no statistical analysis of the difference was conducted because of the small sample size. Overall, there was no loss in visual acuity reported in any treatment group during follow-up in all 3 studies. According to descriptive safety statistics, the frequencies of ocular and nonocular adverse events seemed to be slightly higher among secukinumab groups versus placebo across the 3 studies [13] (Table 1).

### 2.2. DE-109 (Sirolimus)

*Sirolimus* (Santen Pharmaceutical, Osaka, Japan) is a macrolide antibiotic produced naturally by* Streptomyces hygroscopicus, *isolated in soil samples from Easter Island. Although originally developed as an antifungal agent, sirolimus has a potent immunosuppressive and antineoplastic activity that depends upon its binding to specific cytosolic proteins (immunophilins) to generate an immunosuppressive complex (RAPA : FKBP). FKBP-12 is the most relevant immunophilin that inhibits the activation of the mammalian target of rapamycin (mTOR) resulting in the suppression of the cytokine driven T-cell proliferation by blocking and inhibiting several signal transduction pathways (phosphorylation and activation of p70-S6 kinase1 and phosphorylation and inactivating 4E-BP1) [7]. The inhibition of the proliferation of B-cell lymphocytes and IL-2, IL-4, and IL-5 represents other additional immunomodulatory effects of rapamycin.

Clinically, the safety profile of this agent has been studied in other ocular conditions including dry eye syndrome, age-related macular degeneration (AMD), and diabetic macular edema (DME) [14, 15]. Initial studies for uveitis reported that systemic sirolimus was effective in the majority of refractory NIU cases, improving the signs and symptoms of inflammation and reducing the steroid burden. However, the systemic/intravenous route of administration was associated with side effects and/or failure to control uveitis in some patients [16, 17]. The Sirolimus as Therapeutic Approach to Uveitis (SAVE) study evaluated the safety end efficacy of sirolimus administered as a subconjunctival or intravitreal injection in patients with NIU results of this study did not find statistically significant differences in bioactivity between the two study groups at month 6, with both subconjunctival or intravitreal injections showing an improvement of two steps or more in vitreous haze in approximately 40% of the patients [18]. Other clinical trials, including Intravitreal Sirolimus as Therapeutic Approach to Uveitis—Phase 2 (SAVE-2), which is being coordinated by the Ocular Imaging Research and Reading Center at the Truhlsen Eye Institute of the University of Nebraska Medical Center, and The Study Assessing Double-masked Uveitis Treatment (SAKURA), will help to establish the long-term safety and efficacy of local ocular formulation of sirolimus in the future (Table 1).

### 2.3. XOMA 052 (Gevokizumab)

Gevokizumab (XOMA Corporation, Berkley, CA, USA) is a recombinant humanized IgG2 antibody that binds strongly to Interleukin-1*β* (IL-1*β*), thereby preventing activation of the IL-1 receptor [9]. The chronic inflammation in islet cells in patients with type 2 diabetes has been associated with the pathological activation of (IL)-1. A phase 2 study was conducted in 2007 in order to evaluate the safety and biological activity of gevokizumab in patients with type II diabetes. Results of this study showed a significant decrease in C-reactive protein (CRP) and an improvement in glycemic control [19].

A pilot study conducted by Gül et al. in 2012 showed that the recombinant, humanized anti-IL1*β* antibody, XOMA 052, incited a rapid and sustained reduction in inflammation in seven refractory NIU (Adamantiades-Behçet disease) patients. This effect was observed without the need to increase the dose of corticosteroids, despite the discontinuation of other immunomodulatory therapies [20].

Following the results of the initial study, three phase III studies, EYEGUARD-A (for patients with active disease), EYEGUARD-B (for patients with Adamantiades-Behçet's disease), and EYEGUARD-C (for patients with controlled disease), have been initiated [21]. In these studies, subjects receive three monthly injections of gevokizumab (60 mg) followed by an extended assessment phase of the study that will last 36 weeks after completion of the study. The primary outcome is the number of participants with at least two-step reduction in vitreous haze or a reduction to zero in scleral inflammation before or at week 16 (Table 1).

In addition, a phase II open label clinical trial in patients with active noninfectious anterior scleritis is also being conducted with gevokizumab [22].

### 2.4. ESBA105

ESBA105 (Alcon Research, Hünenberg, Switzerland) is a topically administrated antibody fragment against TNF-*α* [23]. In 2009, Ottiger et al. discovered that, even without the use of therapeutic enhancers, it could penetrate into the anterior and posterior chambers at therapeutic levels by translimbal/intrascleral migration [24].

Clinically, the safety and the efficacy of topical administration of ESBA105 were reported in a study of 57 patients who were scheduled for surgery (cataract or vitrectomy); the study reported that topical administration of ESBA105 rapidly achieved high intraocular levels, maintaining a favorable safety and tolerability profile [25]. A pilot study of ESBA105 applied hourly followed by dose tapering was completed in patients with acute anterior uveitis; however, the results of this study are currently not available [26].

### 2.5. Abatacept (Orencia)

T-cell antigen CD28 provides a costimulatory signal needed for T-cell activation; such cascade results in T-cell proliferation and secretion of several lymphokines including interleukin-2 (IL-2). CD28 signaling is triggered by its counter receptors, CD80 and CD86, which are expressed on antigen-presenting cells (APC). Orencia (Bristol-Myers Squibb Company, New York, USA) is a CTLA4-IgG fusion protein that targets CD80/CD86 and consequently blocks T-cell activation [10].

Abatacept has been used in Th-1 mediated diseases such as psoriatic arthritis, juvenile idiopathic arthritis (JIA), and RA [10, 27]. In 2010, Zulian et al. found that Orencia initiated and sustained well-tolerated improvement in refractory cases of psoriatic and JIA-associated anterior uveitis [28]. An open label phase II uveitis study is currently recruiting patients with refractory and vision-threating uveitis [29].

### 2.6. Tocilizumab (Actemra; Roche, Nutley, New Jersey, USA) and Sarilumab (Regeneron Pharmaceuticals, Inc., Tarrytown, NY, USA, and Sanofi, Paris, France)

Interleukin-6 (IL-6) is a pleotropic cytokine produced by T-cells, B-cells, monocytes, fibroblasts, synovial cells, and endothelial cells. It has a wide range of biological activities and is a key player in the pathogenesis of numerous inflammatory disorders such as RA. IL-6 binds to either a transmembrane receptor (mIL-6R) or to a soluble receptor (sIL-6R) formed by the proteolytic cleavage of mIL-6R. After binding to the receptor, IL-6 recruits two molecules of the transducing glycoprotein (gp130) involved in the down-stream signaling process. Signaling by the sIL-6R is a key feature in the pathophysiology of autoimmune diseases and chronic inflammation rather than the mIL-6R. Neutralizing monoclonal antibodies against this pathway are currently under investigation [11].

Tocilizumab (Actemra; Roche, Nutley, New Jersey, USA) and sarilumab (Regeneron Pharmaceuticals, Inc., Tarrytown, NY USA) are humanized antihuman monoclonal IgG1 antibodies synthesized by recombinant DNA technology that target both IL-6 receptors, thereby blocking the proinflammatory effects of IL-6 [11, 12]. TCZ is currently approved in the USA for RA, particularly in treatment refractory cases. The STOP-UVEITIS study, a multicentered clinical trial investigating the safety, efficacy, and bioactivity of different doses of TCZ in patients with NIU, has been initiated in 2012 in the US and is being coordinated by the Ocular Imaging Research and Reading Center at the Truhlsen Eye Institute of the University of Nebraska Medical Center. In addition, a multicentered study investigating the efficacy and safety of sarilumab in patients with NIU (the SATURN Study) is also currently underway at various sites in Europe and United States. The SATURN Study is sponsored by Sanofi in collaboration with Regeneron Pharmaceuticals (Table 1).

### 2.7. EGP-437 (Iontophoretic Dexamethasone Phosphate)

EGP-437 (Eyegate Pharmaceuticals, MA, USA) is a dexamethasone phosphate solution that is delivered to the eye via iontophoresis, a technique first reported in 1943 by von Sallman et al. Iontophoresis consists of applying a current in a controlled manner, by an ocular applicator, for producing ions (hydroxide or hydronium) that drive the drug molecule noninvasively into the anterior and posterior segments of the eye, thereby minimizing the systemic distribution of the drug. Dexamethasone phosphate is a dexamethasone prodrug that is highly water soluble with a buffering ability necessary for iontophoresis [8].

Clinically, EGP-437 has been shown to have prolonged duration of action and has proved to be significantly more effective compared to other delivery routes, such as the topical and subconjunctival route [8, 30]. In 2012, Cohen et al. in a phase I/II study reported that EGP-437 was well tolerated and extremely effective, achieving anterior cell chamber scores of 0 within 28 days after just one treatment in 60% of participants with noninfectious anterior uveitis [8]. Based on these findings, a phase III study comparing ECGP-437 (4-mA/min) with topical prednisolone acetate (1%) to treat noninfectious anterior uveitis was initiated and has been completed recently; the primary outcome in this study will be the percentage of patients with an anterior chamber score of 0 at day 14.

Beyondthe studies on anterior uveitis, a pilot study evaluating the safety of EGP-437 in patients with anterior scleritis has been conducted. Study subjects were randomized to receive either EGP-437 or sham treatment. Dose-limiting toxicity was the primary outcome of this study. The results of this study are awaited [31].

### 2.8. LX211 (Voclosporin)

Voclosporin (Lux Biosciences, Jersey City, NJ, USA) is an orally active next-generation calcineurin inhibitor with potent immunosuppressive activity. Inside the lymphocyte, this molecule forms a complex with immunophilins consequently inhibiting calcineurin. This action prevents the translocation of the cytoplasmic component of the activated T-cells to the nucleus, resulting in impaired transcription of the genes encoding IL-2, a molecule essential for T-cell proliferation and other inflammatory lymphokines [32].

Voclosporin has a structure that is similar to cyclosporine-A, except for a modification in the amino acid-1 residue, which gives the molecule a higher binding affinity for calcineurin and a more predictable pharmacokinetic profile [32]. These characteristics allowed this agent to be an invaluable immunosuppressant in organ transplantation and other autoimmune conditions such as RA and psoriasis [33]. During the past few years, attention has been gained on Th-1 mediated conditions like dry eye syndrome and uveitis. The Lux Uveitis Multicenter Investigation Clinical Program (LUMINATE) was developed to demonstrate the usefulness of voclosporin in patients with active or quiescent posterior uveitis or active anterior uveitis. The results of this study in active posterior uveitis demonstrated a reduction in the vitreous haze in 50% of patients and prolonged the time to recurrence by twofold, while in quiescent uveitis, it reduced the frequency of exacerbations by 50%. In all the study groups, the reduction in the burden of oral prednisolone doses to ≤5 mg/d was reported in 96%–98% of the patients. The results for this drug have so far been comparable to current therapeutic options, with the added benefit of a better safety profile and possibly a better compliance due to its oral route of administration. However, a second phase III trial did not show a statistically significant difference between the placebo and disease groups. No additional studies are planned at this time to evaluate this agent further.

## 3. Conclusion

The management approaches for patients with uveitis are protean and challenging, given the complexity of the pathophysiology of the disease. Clinical recommendations for the treatment of uveitis include a no tolerance policy for any degree of inflammation together with an acceptable dose of corticosteroids (<7.5 mg/day). Such therapeutic principles and algorithm have led to an extensive search for novel immunomodulatory therapies (IMT), in terms of the mechanism of actions or mode of delivery, that would halt or reduce the degree of inflammation in patients with uveitis and, therefore, provide control of the disease and reduce the need for steroid therapy. However, in a number of patients treated with IMT, the treatment is either suboptimal or causes undesirable side effects. An increased understanding of the human immune system in recent times has led to the development of potentially new agents that target the disease pathways in a more effective manner, thereby helping to combat this sight-threatening disease. It is hoped and expected that these potential pharmacologic agents may be used in combination, even with low dose corticosteroids, to provide multimodal and multitargeted control of the inflammatory process.

## Figures and Tables

**Table 1:** Clinical trials for emergent therapies in noninfectious uveitis.

Drug	Mechanism of action	Study name phase	Sample size	Intervention	Results
AIN457 secukinumab (SK)	Fully humanized antibody blocks IL-17A [6]	SHIELD Phase III	118	(i) SK 300 mg s.c. at baseline, week 1 and week 2 (loading phase), and then every 2 weeks (ii) SK 300 mg s.c. loading phase and then monthly (iii) Placebo s.c. loading phase and then every 2 weeks	(i) Greater reduction in mean total postbaseline composite immunosuppressive medication (ISM) in the SK group compared with placebo (*P* = 0.047) (ii) No statically significant differences in change in BCVA between the SK group and placebo (iii) Median decrease in vitreous haze was similar among treatment groups
		INSURE Phase III	31	(i) SK 300 mg s.c. loading phase and then every 2 weeks (ii) SK 300 mg s.c. loading phase and then monthly (iii) SK 150 mg s.c. loading phase and then monthly (iv) Placebo s.c. loading phase and then every 2 weeks	(i) No major differences in mean change in vitreous haze from baseline to week 28 in all groups (ii) ISM score 0.0 for all SK groups, 1.83 for placebo (iii) No loss in BCVA in all the groups (iv) No apparent dose-response relation for the incidence of AEs (adverse events)
		ENDURE Phase III	125	(i) SK 300 mg s.c. loading phase and then every 2 weeks (ii) SK 300 mg s.c. loading phase and then monthly (iii) SK 150 mg s.c. loading phase and then monthly (iv) Placebo s.c. loading phase and then every 2 weeks	(i) No statistically significant differences between all the groups in the time of first recurrence of uveitis (ii) Composite ISM score is similar across all the groups

DE-109 sirolimus	mTOR inhibitor [7]	SAVE Phase I	30	(i) Intravitreal sirolimus: 325 *µ*g at days 0, 60, and 120 (ii) Subconjunctival injection: 1320 *µ*g at days 0, 60, and 120	(i) Did not find statistically significant differences between the two study groups at month 6 (ii) improvement of two steps or more of vitreous haze in 40% of the patients
		SAVE-2 Phase II	30	(i) Intravitreal sirolimus 440 *µ*g at baseline and months 1, 2, 3, 4, and 5 and then prn after month 6 (ii) Intravitreal sirolimus 880 *µ*g at baseline and months 2 and 4 and then prn after month 6	Recruiting
		SAKURA Phase III	500	(i) Sirolimus low dose (44 *µ*g) intravitreal (ii) Sirolimus medium dose (440 *µ*g) intravitreal (iii) Sirolimus high dose (880 *µ*g) intravitreal	Recruiting

EGP-437 iontophoresis dexamethasone phosphate	Glucocorticoid receptor antagonist [8]	Phase I/II	40	(i) 1.6 mA-min (ii) 4.8 mA-min (iii) 10 mA-min (iv) 14 mA-min	(i) By day 28, 40 patients (60%) achieved an anterior chamber cell score of zero (ii) 1.6 mA-min was the most effective dose (iii) Intraocular pressure and BCVA remained stable throughout the study

XOMA 052 gevokizumab	Recombinant humanized anti-IL1*β* antibody [9]	EYEGUARD A Active Uveitis Phase III		(i) Placebo drug s.c. (ii) Group 1 gevokizumab s.c. (iii) Group 2 gevokizumab s.c.	Recruiting
		EYEGUARD C Controlled Uveitis Phase III		(i) Placebo drug s.c. (ii) Dose 1 gevokizumab s.c. (iii) Dose 2 gevokizumab s.c.	Recruiting

Abatacept (Orencia)	CD28 inhibitor [10]	Abatacept in the Treatment of Noninfectious Uveitis Phase II	20	(i) Abatacept 10 mg/kg for the first 6 months (ii) At month 6 randomization to receive either 5 mg mg/kg or 10 mg/kg	Recruiting

Tocilizumab	IgG1 recombinant humanized antihuman monoclonal antibodies that target IL-6 receptors [11, 12]	STOP Uveitis Phase I-II	36	(i) Tocilizumab 4 mg IV, 6 monthly doses, and then prn after month 6 (ii) Tocilizumab 8 mg IV 6 monthly doses, and then prn after month 6	Recruiting
Sarilumab		Study to Analyze Sarilumab in Noninfectious Uveitis Phase II	57	(i) Sarilumab s.c. every 2 weeks up to 50 weeks (ii) Placebo s.c. every 2 weeks up to 50 weeks (iii) In both groups prednisone as single therapy or in combination with methotrexate are continued	Recruiting
